# Expression of Glucose Transporters 1 and 3 in the Placenta of Pregnant Women with Gestational Diabetes Mellitus

**DOI:** 10.3390/life13040993

**Published:** 2023-04-12

**Authors:** Waleed Aldahmash, Abdel Halim Harrath, Khaldoon Aljerian, Yasser Sabr, Saleh Alwasel

**Affiliations:** 1 Zoology Department, College of Science, King Saud University, P.O. Box 2455, Riyadh 11451, Saudi Arabia; 2 Pathology Department, College of Medicine, King Saud University, Riyadh 11451, Saudi Arabia; 3 Obstetrics and Gynaecology Department, College of Medicine, King Saud University, Riyadh 11451, Saudi Arabia

**Keywords:** glucose transporter, placenta, pregnant women, gestational diabetes mellitus, apoptosis, birth weight

## Abstract

Background: The annual prevalence of gestational diabetes mellitus—characterized by an increase in blood glucose in pregnant women—has been increasing worldwide. The goal of this study was to evaluate the expression of glucose transporter 1 (GLUT1) and glucose transporter 3 (GLUT3) in the placenta of women with gestational diabetes mellitus. Methods: Sixty-five placentas from women admitted to the King Saud University Medical City, Riyadh, Saudi Arabia, were analyzed; 34 and 31 placentas were from healthy pregnant women and women with gestational diabetes, respectively. The expressions of GLUT1 and GLUT3 were assessed using RT-PCR, Western blotting, and immunohistochemical methods. The degree of apoptosis in the placental villi was estimated via a TUNEL assay. Results: The results of the protein expression assays and immunohistochemical staining showed that the levels of GLUT1 and GLUT3 were significantly higher in the placentas of pregnant women with gestational diabetes than those in the placentas of healthy pregnant women. In addition, the findings showed an increase in apoptosis in the placenta of pregnant women with gestational diabetes compared to that in the placenta of healthy pregnant women. However, the results of gene expression assays showed no significant difference between the two groups. Conclusions: Based on these results, we conclude that gestational diabetes mellitus leads to an increased incidence of apoptosis in the placental villi and alters the level of GLUT1 and GLUT3 protein expressions in the placenta of women with gestational diabetes. Understanding the conditions in which the fetus develops in the womb of a pregnant woman with gestational diabetes may help researchers understand the underlying causes of the development of chronic diseases later in life.

## 1. Introduction

Glucose intolerance that occurs during pregnancy is known as gestational diabetes mellitus (GDM) [1]. When a pregnant woman’s body is incapable of acclimatize to her new environment and her endocrine system becomes unable to create adequate insulin, gestational diabetes mellitus develops [2]. Whalen and Taylor indicated that gestational diabetes is caused by hormonal changes that occur in the body during pregnancy, which causes insulin resistance [3]. The prevalence of GDM has been rising over time, which may be attributed to relevant factors such as maternal age and rising obesity rates, among others [4,5,6]. It is estimated that between 7% and 27% of pregnant women worldwide have gestational diabetes [7]. According to statistical studies, the prevalence of GDM in Saudi Arabia was approximately 10.8% during the period 2000–2009, and this percentage increased to 18.2% during 2010–2018 [8]. GDM is characterized by a high level of glucose in the pregnant woman’s blood during pregnancy. GDM results in a number of detrimental outcomes for the mother and baby, including an increased likelihood of caesarean section, preeclampsia, gestational hypertension [9], fetal macrosomia [10], neonatal hypoglycemia, hyperbilirubinemia, and hyperinsulinemia [11,12,13], as well as potential long-term outcomes [14]. GDM has been shown to have long-term effects on the health of women and that of their children [15]. Studies show that mothers who have GDM are more likely to develop type 2 diabetes mellitus (T2DM), and their children are more likely to become obese as children [12,16], and have five times the risk of developing type 2 diabetes compared to the children of healthy women [17,18].

The placenta is a temporary, vital organ that only grows during pregnancy. The placenta is crucial to the life of the developing fetus inside the mother’s womb, as it facilitates the transport of needed nutrients and oxygen from the mother’s circulation to the fetal circulation. Additionally, it releases a variety of hormones that assist in maintaining pregnancy until delivery. Gluconeogenesis in the fetus is very low [19]. Consequently, the growth of the fetus is directly dependent on the availability of maternal nutrients and the placenta’s ability to deliver these nutrients into the fetus [20]. GDM affects the placenta in several ways, including the morphology of the placenta, as it leads to an increase in its weight, thickness, and surface area [21,22,23]. On the other side, GDM induces maternal vasculopathies in the placenta, such as decidual vasculopathy, syncytial knots, and calcification, in addition to fetal vasculopathies such as chorangiosis, villous fibrinoid necrosis, and edema [24]. Furthermore, GDM changes DNA methylation in the placenta [25].

A placental transporter facilitates the flow of nutrients to the fetal blood vessels. Glucose has been shown to be the main source of energy for the growth of both the placenta and the fetus [26]. Glucose transport in the placenta is mediated by members of the glucose transporter (GLUT) family, which aid in the diffusion of glucose along a concentration gradient [27]. The GLUT family has 12 members, of which the GLUT1 and GLUT3 isoforms are the major placental glucose transporters in humans. As expected, these isoforms are widely expressed during early pregnancy and at term [28]. GLUT1 and GLUT3 are present in the microvilli and basement membrane of the syncytiotrophoblast, although they are also expressed in the cytotrophoblast and endothelium of blood vessels [29,30].

Numerous investigations have been conducted on GLUTs in the placenta of women with type 1 or 2 diabetes or GDM. In the case of GDM, there are different conditions for controlling high glucose due to the disease appearing for the first time, and the response of the pregnant woman to the disease varies depending on the medical recommendation. Some pregnant women with GDM mange it by diet, while others use exercise or insulin or other medications [20].

Given that women with GDM experience an increase in blood glucose, our aim in this study was to evaluate the gene expression and protein expression of GLUT1 and GLUT3 in the placentas of women with GDM who did not have their condition under control.

## 2. Materials and Methods

### 2.1. Study Design

This study was conducted at King Saud University Medical City (KSUMC), Riyadh, Saudi Arabia. Placentas from Saudi women who had been admitted to the obstetrics and gynecology unit of the hospital between January and June 2019 were obtained. GDM was diagnosed according to the criteria of the International Association of Diabetes and Pregnancy Study Groups (IADPSG), during 24–28 weeks of gestation [31]. The study’s pregnant participants were separated into two groups: one with gestational diabetes and the other with normal blood glucose levels, based on the outcomes of the oral glucose tolerance test (OGTT). This test involves collecting a blood sample from pregnant women after they have fasted for eight hours. After that, they were given 75 g of glucose syrup to consume, and then the blood sample was collected again from them an hour and two hours later. The IADPSG criteria state that a pregnant woman has gestational diabetes if her blood glucose level is ≥5.1 mmol/L (92 Mg/dL) after 8 h of fasting, or/and ≥10 mmol/L (180 Mg/dL) an hour after taking glucose syrup, or/and ≥8.5 mmol/L (153 Mg/dL) after two hours.

The age of pregnant, healthy women participating in this study ranged between 18 and 40 years, whereas the age of women with GDM ranged between 23 and 40 years old. In addition, although the weight of pregnant, healthy women who participated varied from 49 to 100 kg, the weight of women with GDM ranged between 60 and 118 kg. The height of pregnant women participating in this study was measured to calculate their body mass index (BMI) according to the following formula: BMI = weight (kg)/height (m^2^) [32]. The BMI ranged between 20 and 40 kg/m^2^ in the pregnant healthy women and between 24 and 44 kg/m^2^ in the pregnant women with GDM.

The current study excluded any pregnant women with GDM who used medications to control their diabetes and pregnant women with GDM who make a diet. The placentas from women with twin deliveries, hypertension, type 1 or type 2 diabetes, or other chronic diseases were excluded. The pregnant women who gave birth through caesarean section were also excluded from the current study. The placentas from healthy pregnant women were considered as the control group.

### 2.2. Collection of Placentas

Full-term placentas were harvested from a singleton pregnancy after delivery. Sixty-five placentas were obtained; thirty-four from healthy pregnant women and thirty-one from women diagnosed with GDM. All participants in this study provided their consent to be involved as well as having their placentas collected. The King Saud University Medical City’s Institutional Review Board approved the study’s conduction as no IRB: E17-2729. The fresh placentas were washed with a normal saline solution to remove any blood clots. The placentas were weighed without membranes or umbilical cord to the nearest 10 g using a digital balance, and the length and width were recorded. In addition, the volume and thickness of the placenta were calculated. The length and diameter of the umbilical cord were also measured. Additionally, immediately upon delivery, the babies’ measurements were recorded.

To evaluate gene expression, samples of the placenta were preserved in RNA later and in 10% neutral buffered formalin (NBF 10%) for immunohistochemical and apoptosis investigations. In the interest of evaluating protein expression, samples were also briefly frozen in liquid nitrogen before being maintained at −80 °C.

### 2.3. Gene Expression Estimation

Total RNA was extracted and purified from fresh placental tissue (50 mg) using RNeasy mini kit (Qiagen, Hilden, Germany) according to the manufacturer’s protocol. The concentration of RNA was measured using a Nano Drop 2000/2000 c Spectrophotometer (Thermo Fisher Scientific, Waltham, MA, USA). The total RNA sample (1 mg/sample) was used to generate cDNA using a High-Capacity cDNA Reverse Transcription Kit (Applied Biosystems, Carlsbad City, CA, USA) according to the manufacturer’s protocol. Quantitative real-time PCR was performed using a kit from Applied Biosystems, Life Technologies, Carlsbad, CA, USA with SYBR^®^ Green PCR master mix. The forward primer, 5′-ATGGCGGGTTGTGCCATA-3′, and the reverse primer, 5′-ATAGGACATCCAGGGTAGCTGCTCC-3′, were used to measure the expression levels of GLUT1. The forward primer, 5′-CAGGCACACGGTGCAGATAG-3′, and the reverse primer, 5′-GCAGGCTCGATGCTGTTCAT-3′, were used to measure the expression levels of GLUT3. The forward primer, 5′-CTGGCACCCAGCACAATG-3′, and the reverse primer, 5′-GCCGATCCACACGGAGTACT-3′, were used to measure the expression levels of β-actin. The relative expression level of each sample was standardized to the level associated with β-actin (Humanizing Genomics, Macrogen, Korea). The 2^−ΔΔCT^ method was used to calculate the relative expression of the target genes [33].

### 2.4. Protein Expression

Proteins were extracted from placental tissue samples using a mammalian protein extraction reagent (Thermo Fisher Scientific), and the Bradford assay was used for protein quantification. After denaturing the proteins by boiling in a 2× Laemmli buffer at 95 °C for 5 min, 20 µg of protein was separated using 12.5% sodium dodecyl sulphate–polyacrylamide gel electrophoresis (SDS-PAGE). The proteins were blotted from the gel to the polyvinylidene difluoride (PVDF) membrane. The membrane, with the primary antibody, was incubated in a shaker overnight at 4 °C. Fetal bovine serum (5%) was used as a blocking agent for 1 h at room temperature, and the membrane was incubated with the secondary antibody for 1 h at room temperature. An ECL kit (Thermal Fisher Scientific) was used to detect the signals. Lastly, the Gel Doc XR+System (BIO-RAD. No: 731BR01868) was used to quantify immunoblotting, and the mean value of the control group was used to determine the relative protein expression.

### 2.5. Antibodies

Rabbit anti-GLUT1 polyclonal antibody (Biorbyt, orb157188) and rabbit anti-GLUT3 polyclonal antibody (Biorbyt, orb10727) were used as the primary antibodies. The goat anti-rabbit IgG H&L (HRP) (Abcam, ab6721) was used as the secondary antibody. With respect to the reference gene, the mouse anti-β-actin antibody (Sigma Aldrich, A5441) was used as the primary antibody, with goat anti-mouse IgG H&L (HRP) (Abcam, ab6789) considered the secondary antibody. All antibodies were diluted according to the manufacturer’s instructions.

### 2.6. Immunohistochemical (IHC) Staining

Tissue samples from the placenta were cut from the maternal side to the fetal side. The samples were taken from the central and marginal regions of the placental disc. The samples were fixed in 10% of NBF and underwent standard protocols for dehydration, clearing, infiltration, and paraffin embedding. The tissue was cut into 2 to 3 μM sections. Tissue sections were stained according to the manufacturer’s instructions (Novolink Max Polymer Detection System, Product No: RE7280-K, Leica). The tissue sections were deparaffinized with xylene, rehydrated with a descending series of ethanol, and then incubated with peroxidase for 5 min. Subsequently, the tissue sections were incubated with a protein block for 5 min. Antibodies that were reactive to human GLUT1 (1:500, Biorbyt, orb157188) and GLUT3 (1:500, Biorbyt, orb10727) were applied overnight at 4 °C. A control slide without the primary antibody (-ve control) was created to confirm the validity of the work and the efficiency of the primary antibodies used. The tissue sections were then incubated with post primary for 30 min, followed by incubation with Novolink polymer for 30 min. The sections were then stained with 3,3′-diaminobenzidine (DAB) for 5 min and counterstained with hematoxylin for 5 min. The prepared tissue sections were photographed using a light microscope (Olympus BX51 connected with an Olympus DP72 camera, Tokyo, Japan). The Aperio-CS2 Scan Scope slide scanner (Leica Biosystems, Vista, CA, USA) was used to quantify DAB intensity.

### 2.7. Apoptosis

The terminal deoxynucleotidyl transferase dUTP-mediated nick-end labeling (TUNEL) assay was used to estimate the extent of apoptosis. The blocks of placenta samples were cut in 3 µm in thickness and loaded into slides. The tissue sections were dried and then kept on a hot plate for 10 min at 60 °C. Tissue sections were immersed in xylene twice (10 min each) and then rehydrated in alcohol in a descending series of concentrations (100%, 95%, 80%, 70%, 50%) for 5 min for each concentration. Distilled water was used to wash the tissue sections. The tissue sections were then immersed in phosphate-buffered saline, followed by incubation with proteinase K for 15 min. Next, 0.1% Triton X-100 with sodium citrate was used to permeabilize the tissue sections. The tissue sections were then incubated with 0.3% pepsin in HCl (pH 2) for 5 min at 37 °C. Tissue sections were immersed in citrate buffer and placed in a microwave at 750 W for 30 s, after which the tissue sections were washed twice with phosphate-buffered saline. TUNEL staining was performed using the In Situ Cell Death Detection kit (TMR-red, Roche Diagnostics, Mannheim, Germany) following the manufacturer’s instructions. To induce DNA fragmentation, a positive control section was treated with recombinant DNase-I for 10 min at room temperature. Additionally, terminal deoxynucleotidyl transferase (TdT) was used to treat some tissue sections as negative controls. Hoechst dye was then used to stain all tissue sections, which were then washed in Tris-EDTA (TE) buffer, pH 8.0 and mounted in a 50% glycerol/TE solution. The prepared tissue sections were photographed using confocal microscopy (Olympus BX61-32FA1-S08 microscope with fluorescence equipment; Olympus, Tokyo, Japan) for morphologic evaluation.

### 2.8. Statistical Analysis

SPSS software version 26 (IBM Inc., Armonk, NY, USA) was used for statistical analysis of the data. The data are reported as mean ± standard deviation (SD). The independent t-test was performed to compare the GDM group with healthy pregnant group. The plots were generated using GraphPad prism software, version 9. Differences between the two groups were considered significant at *p* < 0.05.

## 3. Results

The characteristics of the pregnant women in this study (34 healthy pregnant women and 31 pregnant women with GDM) as well as the pregnancy outcomes are shown in Table 1. The maternal age, weight, and body mass index were significantly higher in pregnant women with GDM compared to healthy pregnant women, and the results of the oral glucose tolerance test (OGTT) were higher in the GDM women at all stages. Regarding maternal height, the results showed no difference between the healthy pregnant and GDM pregnant women. The mean birth weight and thigh circumference of the newborns from the GDM group of women were significantly higher than those of the newborns from the healthy pregnant group of women. The placental weight, length, and umbilical cord diameter were all higher in the GDM group compared to those in the control group. However, the gestational age of babies born in the GDM group was lower than that in the control (healthy) group.

**Figure 1:**
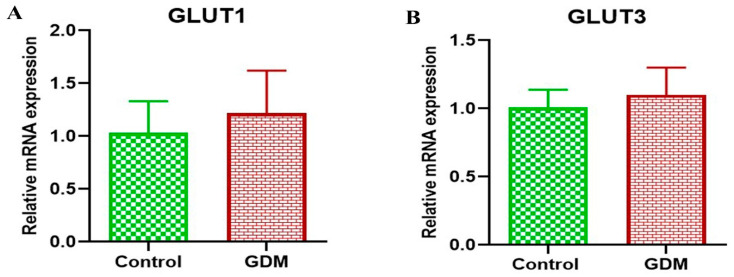
mRNA expression of GLUT1 (**A**) and GLUT3 (**B**). The results show that there was no significant difference between the placentas of pregnant women with gestational diabetes mellitus (GDM) and those of healthy pregnant women.

**Figure 2:**
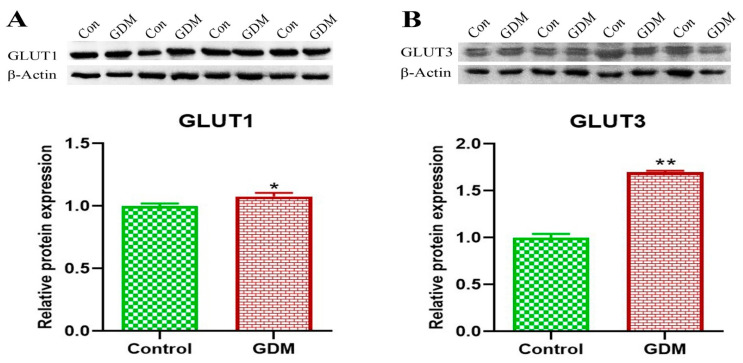
Protein expression of GLUT1 (**A**) and GLUT3 (**B**) on chorionic villi of the placenta. The results showed a significant increase in the protein expressions of GLUT1 and GLUT 3 in the placentas of pregnant women with GDM compared to the corresponding values in the placentas of healthy pregnant women. (*) *p* ≤ 0.05. (**) *p* ≤ 0.01.

**Figure 3:**
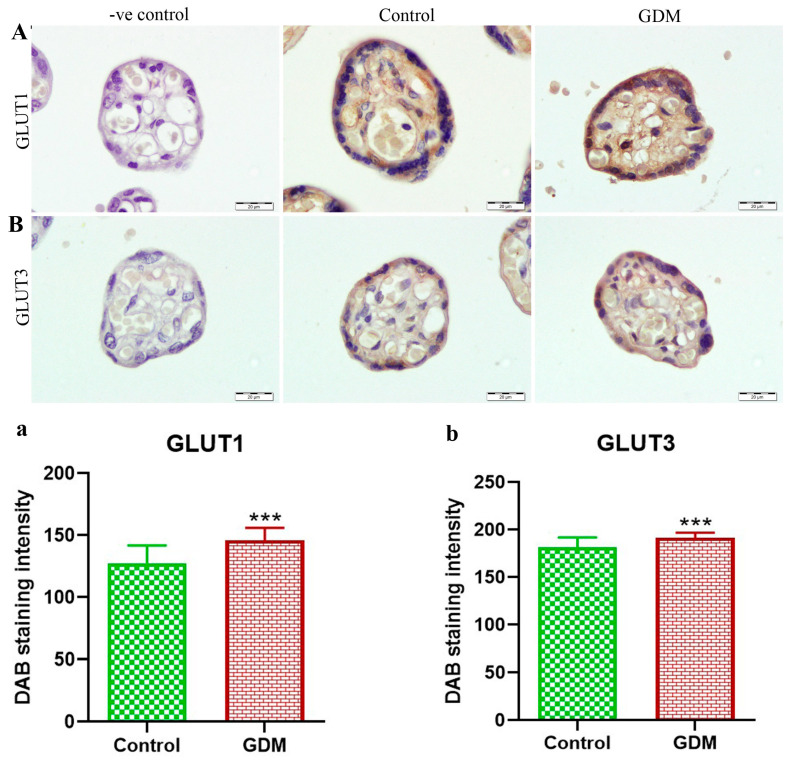
Results of immunohistochemical (IHC) staining of GLUT1 and GLUT3 in placenta sections. (**A**,**B**) IHC images of placenta sections, scale bar 20 µm (-ve control: without primary antibodies). (**a**,**b**) Statistical analysis of IHC results. The results demonstrated that the GLUT1 and GLUT3 were significantly increased in the placentas of pregnant women with GDM compared to healthy pregnant women (***) *p* ≤ 0.001.

**Figure 4:**
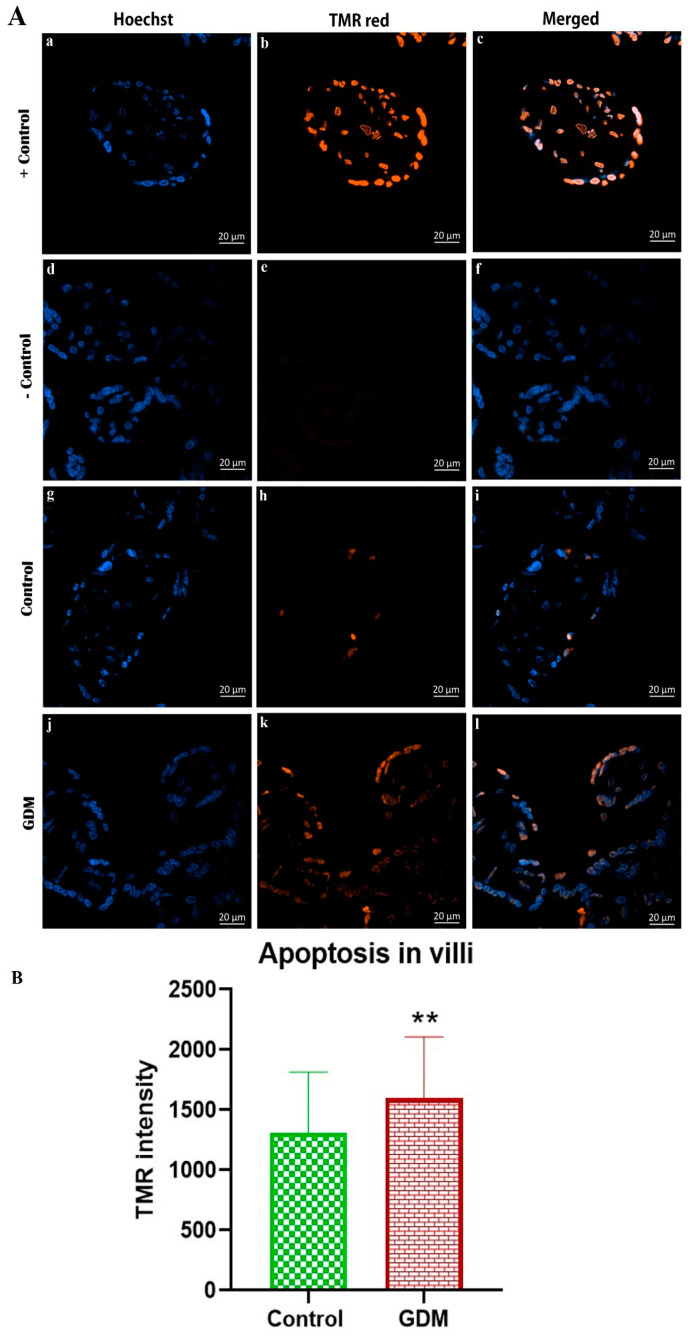
Apoptosis in the placental villi. (**A**) Representative images of apoptosis in placental villi stained with Hoechst and TMR red. Scale bar, 20 μm. (**B**) Apoptosis rate in placental villi. TMR intensity in the villi of placentas of pregnant women with GDM was higher compared to the corresponding values in the healthy pregnant women. (**) *p* ≤ 0.01.

The gene expression levels of GLUT1 and GLUT3 in the placental villi showed no significant difference between the placentas of women with GDM compared to healthy women (Figure 1). By contrast, the protein expression levels for both GLUT1 and GLUT3 were significantly higher in the placentas of women with GDM compared to the placentas of healthy pregnant women (Figure 2). Additionally, the results of the IHC staining (Figure 3) showed that the GLUT1 and GLUT3 protein densities were higher in the placentas of pregnant women with GDM than in the healthy pregnant women. Furthermore, we found an increased incidence of apoptosis in the placental villus cells of the GDM group compared to that in the placental villus cells of the healthy pregnant group (Figure 4).

## 4. Discussion

This study was conducted on Saudi women in the city of Riyadh. The results of the demographic parameters showed an increase in weight, age, and body mass index for mothers with gestational diabetes compared to healthy mothers, indicating a relationship between advancing age and increasing body mass with the possibility of GDM appearing during pregnancy, and this is consistent with studies conducted on Saudi women or non-Saudi women [34,35,36,37].

The gestational age for newborns has decreased in pregnant women with gestational diabetes mellitus according to several previous studies [34,38,39], which agree with the current study, but the gestational age of the newborns in both groups is still at full term. In spite of this, the weight of the newborns and placentas increased in women with GDM, and this was indicated by previous studies [21,34,39,40,41]. Likewise, several investigations revealed a relationship between pregnant women with GDM and macrosomia in newborns [10].

GLUT1 and GLUT3 are the two most common isoforms of GLUT in the human placenta, and they are essential for the transportation of glucose into the fetal circulatory system [30,42,43,44]. In the present study, we investigated the expression of the genes GLUT1 and GLUT3 as well as the corresponding protein densities in the term placentas of healthy pregnant women and pregnant women with GDM.

With respect to GLUT1, we found no appreciable variation in gene expressions, which is consistent with the results of several other studies [26,45,46]. In contrast, and in line with the results of many other studies [26,47,48,49,50], the level of GLUT1 protein (as determined through immunoblotting as well as HIC staining) was higher in the placentas of pregnant women with GDM compared to the healthy pregnant women. Increased GLUT1 in the placenta of pregnant women with GDM may be due to increased glucose availability in the mother’s circulation and a corresponding increase in the consumption of glucose in the placenta [48]. It is, therefore, reasonable to assume that a rise in the mother’s blood glucose level may also elevate the level of glucose in the blood of the fetus, and, as a result, the gene expression of glucose transporters is increased in both the placenta and fetus [51].

Similar to the results for GLUT1, there was no significant difference between the expression of the GLUT3 gene in the placentas of the women with GDM and healthy pregnant women; however, the level of GLUT3 protein was higher in the placentas of the GDM group than that in healthy pregnant women. The results of previous studies have indicated that the structure and function of the placenta is altered in women with GDM [24,52]. It is possible to hypothesize that placental tissue abnormalities, which have been shown to impair insulin signaling and, consequently, glucose transport, may contribute to long-term negative effects in both the mother and child, and these effects depend primarily on the physiology of the mother [53].

The glucose necessary for fetal development is supplied by the blood circulation of the mother. Glucose is delivered from the mother to the fetus by glucose transporters through facilitated diffusion. The capacity and activity of the basement and microvillus membranes of the cytotrophoblast to supply glucose and the glucose transporter density all play a role in controlling glucose transport in the placenta [54]. Placental glucose metabolism, including gluconeogenesis, glycolysis, and blood flow from the womb to the placenta, can also have an impact on transplacental glucose transfer [55]. Song and colleagues reported that pregnant women with GDM experience an abnormal glucose metabolism as a result of the overexpression of GLUT1 in the placenta, which is brought on by a decrease in the levels of miR-9 and miR-22 [56].

The relationship between GLUT1 expression and the concentration of glucose in the mother’s blood circulation has been shown in previous studies [26,57]. An increase in the concentration of glucose in the mother’s blood leads to an increase in GLUT1 expression to a certain extent within the physiological concentration range, but the continued increase in the mother’s blood glucose leads to saturation of GLUT1, which triggers the transfer of glucose to the fetal blood circulation. Thus, the fetus receives a surplus of calories, which can supply energy to the developing body or may be stored as fat—which would account for the increase in the birth weight of GDM infants. This is consistent with the results of the current and previous studies [24,39,41,58,59,60]. This was demonstrated by the hyperinsulinemia in the newborns’ blood after birth [11,12,13].

The results of our study revealed that there was no significant change in the gene expressions of either GLUT1 or GLUT3 in the placentas of women with GDM compared to healthy women, whereas there was an increase in the protein levels for both GLUT1 and GLUT3. These findings indicate that there is no difference in the transcription of GLUT1 and GLUT3, but there may be a defect in the translation of the mRNA. Additionally, these results may indicate the presence of different pathways in the expression of glucose transporters. Therefore, this study recommends conducting more research to find out the different pathways of glucose transporter expression. Previous studies have shown that the regulation of the amount of GLUT1 in the cell membranes takes place after transcription [61].

Apoptosis is an important physiological process for placental homeostasis. An imbalance in apoptosis may affect placental functions. The results of the present study showed an increase in the rate of apoptosis in the placenta of pregnant women with GDM compared to healthy pregnant women, and this is consistent with findings in previous studies that were conducted on human [62,63,64] or animal models [65]. This change in the degree of apoptosis may be due to the increase in the level of sugar in the mother’s blood, given that it has been shown that there is a relationship between the increase in sugar in the blood of pregnant women and an increase in the incidence of apoptosis in the placenta [66].

The placenta influences both the metabolism of the pregnant woman and the development of the fetus, and therefore genetic functional alterations (also known as epigenetics) of the placenta are likely to lead to pregnancy complications and disease susceptibility for the mother and her fetus [67]. These changes involve several mechanisms, such as DNA and RNA methylation, chromatin remodeling, modification of histones, and the expression of noncoding RNAs. Thus, the placenta has its own genetic programming during pregnancy [68] and may undergo genetic changes resulting from gestational diabetes [69]. Therefore, most studies related to genetic modifications of the placenta have focused on the study of DNA methylation [70] and mRNA gene expression [71], which may alter the placental function and its gene expression.

## 5. Conclusions

The results of our study of GLUT1 and GLUT3 expression in the placentas of women with GDM underscore the dependence of the health of the fetus on the blood physiology of the mother, in this case the level of glucose. We found that the incidence of apoptosis in the placental villi increased in mothers with GDM, and the level of GLUT1 and GLUT3 proteins (as determined by both immunoblotting and IHC) was also altered in the GDM placentas. These results contribute to a better understanding of the conditions in which the fetus lives in the womb of a pregnant woman with GDM, which may help researchers identify the underlying causes of chronic diseases that appear later in life.

## Figures and Tables

**Table 1:** Anthropometrics of the study population.

		Control (*n* = 34)	GDM (*n* = 31)	*p* Value
		Mean ± SD	Mean ± SD	
**Mothers**	Age (year)	28.2 ± 5.9	32.4 ± 5.3	<0.01
	Height (cm)	158 ± 4.3	157.3 ± 4.3	0.51
	Weight (kg)	73.4 ± 14.5	84.3 ± 14.3	<0.01
	BMI (kg/m^2^)	29.2 ± 5.4	34.1 ± 5.4	<0.01
**OGTT**	Fasting	4.5 ± 0.8	5.2 ± 1.2	0.02
**(mmol/L)**	1-h	7.2 ± 1.8	9.9 ± 1.8	<0.01
	2-h	5.9 ± 1.6	8.9 ± 1.9	<0.01
**Newborns**	Gestational age (week)	39.3 ± 0.9	38.5 ± 1.6	0.02
	Birth weight (g)	3110 ± 344	3338 ± 377	0.02
	Thigh circumference (cm)	15.8 ± 1.2	16.6 ± 1.7	0.04
**Placentas**	Weight (g)	400 ± 58	451.2 ± 87	<0.01
	Length (cm)	18.4 ± 2	19.6 ± 2.3	0.04
	Width (cm)	16.2 ± 1.6	16.2 ± 1.8	0.86
**Umbilical Cords**	Length (cm)	53 ± 9.8	56.1 ± 13	0.19
	Diameter (cm)	1.05 ± 0.18	1.2 ± 0.18	<0.01

OGTT: Oral glucose tolerance test, BMI: Body mass index.

## Data Availability

Not applicable.

## Abbreviations

DAB	3:3′-diaminobenzidine
ECL	Enhanced chemiluminescent
GDM	Gestational diabetes mellitus
GLUT	Glucose transporter
GLUT1	Glucose transporter 1
GLUT3	Glucose transporter 3
IADPSG	International Association of Diabetes and Pregnancy Study Groups
IHC	Immunohistochemical
KSUMC	King Saud University Medical City
NBF	Neutral buffered formalin
OGTT	Oral glucose tolerance test
PVDF	Polyvinylidene difluoride
TdT	Terminal deoxynucleotidyl transferase
TE	Tris-EDTA (Ethylenediaminetetraacetic acid)
TUNEL	Terminal deoxynucleotidyl transferase dUTP nick end labeling
